# Plasmonic Effect of Au Nanoparticles Deposited onto TiO_2_-Impact on the Photocatalytic Conversion of Acetaldehyde

**DOI:** 10.3390/molecules30153118

**Published:** 2025-07-25

**Authors:** Maciej Trzeciak, Jacek Przepiórski, Agnieszka Kałamaga, Beata Tryba

**Affiliations:** Department of Catalytic and Sorbent Materials Engineering, Faculty of Chemical Technology and Engineering, West Pomeranian University of Technology in Szczecin, Pułaskiego 10, 70-322 Szczecin, Poland; maciej.trzeciak@zut.edu.pl (M.T.); jacek.przepiorski@zut.edu.pl (J.P.); agnieszka.kalamaga@zut.edu.pl (A.K.)

**Keywords:** Au-deposited TiO_2_, acetaldehyde decomposition, plasmonic effect

## Abstract

A comparison of two synthesis methods for depositing Au nanoparticles onto TiO_2_ was performed: (1) impregnation with HAuCl_4_ followed by thermal treatment in argon, and (2) magnetron sputtering from a Au disc. The obtained materials were used for acetaldehyde decomposition in a high temperature reaction chamber and ch aracterised by UV-Vis/DR, XPS, XRD, SEM, and photoluminescence measurements. The process was carried out using an air/acetaldehyde gas flow under UV or UV-Vis LED irradiation. The mechanism of acetaldehyde decomposition and conversion was elaborated by in situ FTIR measurements of the photocatalyst surface during the reaction. Simultaneously, concentration of acetaldehyde in the outlet gas was monitored using gas chromatography. All the Au/TiO_2_ samples showed absorption in the visible region, with a maximum around 550 nm. The plasmonic effect of Au nanoparticles was observed under UV-Vis light irradiation, especially at elevated temperatures such as 100 °C, for Au/TiO_2_ prepared by the magnetron sputtering method. This resulted in a significant increase in the conversion of acetaldehyde at the beginning, followed by gradual decrease over time. The collected FTIR spectra indicated that, under UV-Vis light, acetaldehyde was strongly adsorbed onto Au/TiO_2_ surface and formed crotonaldehyde or aldol. Under UV, acetaldehyde was mainly adsorbed in the form of acetate species. The plasmonic effect of Au nanoparticles increased the adsorption of acetaldehyde molecules onto TiO_2_ surface, while reducing their decomposition rate. The increased temperature of the process enhanced the decomposition of the acetaldehyde.

## 1. Introduction

The elimination of volatile organic compounds (VOCs) by photocatalytic methods has recently attracted considerable attention from the scientific community. The reason for this is that these compounds are commonly present in the indoor air and have the potential to increase the risk of Sick Building Syndrome (SBS) in residents who spend a significant amount of time in such spaces [1,2]. Among them, acetaldehyde is the most commonly used model compound for studies. Furthermore, being classified as a suspected carcinogen, acetaldehyde is recommended as a model pollutant in ISO standards for assessing the activity of photocatalytic ceramic materials. Although TiO_2_ is the most widely used material for the photocatalytic air cleaning, various modifications have been attempted to enhance its activity. One of the most promising modifications is doping TiO_2_ with noble metals to increase the visible light response and its chemical reactivity caused by the occurring interfacial charge transfer between metal particles and TiO_2_ [3,4,5,6,7]. Noble metals are commonly utilised as catalysts or co-catalysts in many chemical processes [8,9,10]. In addition, they exhibit a localised surface plasmon resonance (LSPR) effect when exposed to light irradiation at wavelengths within their absorption range. Most of them absorb visible or near-IR light, while Au NPs absorb light in the range of 450–650 nm, depending on their size and shape [11,12,13]. Such properties of Au NPs are considered as crucial for the preparation of photocatalysts active under visible light. The strength of plasmonic effect of Au NPs depends on the particles size distribution and intensity of irradiation [14,15,16]. In general, the LSPR shifts towards longer wavelengths as the size increases, since larger metal NPs require a lower frequency for electromagnetic retardation [17]. Some researchers have reported that the LSPR intensity increases as the size of the Au nanoparticles increases [18]. The LSPR excitation in plasmonic NPs generates intense electric fields near the nanoparticle surface. Photonic energy can be converted into electronic or thermal energy through the decay of surface plasmons via two pathways: radiative decay, which transforms plasmons into photons, and nonradiative decay, which generates electron-hole excitations, also known as “hot spots” [19]. This phenomenon can be beneficial in photocatalytic systems, as these hot carriers can escape from the plasmonic NPs and induce further chemical reactions. Highly energetic hot electrons can be injected directly into the conduction band of a neighbouring semiconductor, thereby enhancing its activity [20]. Such a mechanism carried out under visible light irradiation was presented by Ohtani et al. for acetic acid and 2-propanol photooxidation [11]. First, incident photons are absorbed by the gold particles. Then, an electron is injected from the gold particles into the conduction band of titania, thus reducing molecular oxygen adsorbed on the titania surface. Next, the electron-deficient gold oxidises organic compounds and is subsequently reduced back to its original metallic state [9]. Contrary to visible light activation of Au/TiO_2_, under UV irradiation Au nanoparticles can serve as an electron sink for conduction band electrons generated in light-absorbing TiO_2_, thereby enhancing electron–hole pair separation and extending their lifetimes. It is proposed that such enhanced charge separation occurs through formation of a Schottky junction as a result of the contact between the metal nanoparticle and the semiconductor [19]. Under dark conditions, TiO_2_ doped Au NPs can catalyse the oxidation of acetaldehyde to acetic acid by dissociating O_2_ and forming of O adatoms [21]. In this case, it was observed that acetaldehyde adsorbed on the surface of Au/TiO_2_ as acetate species. The carboxyl groups of acetic acid are strongly bound to the TiO_2_ surface. As a consequence of the intensified conversion of acetaldehyde to acetic acid in the presence of gold nanoparticles, high decomposition of the acetaldehyde was achieved. However, when the acetaldehyde decomposition is conducted on TiO_2_ without Au NPs in a continuous flow reactor, the photocatalyst undergoes deactivation over time [22]. In such cases, the addition of a co-catalyst to TiO_2_ can significantly improve photocatalytic activity and stability of TiO_2_, especially at elevated temperatures [18,19]. In previous studies, we have indicated that acetaldehyde decomposition on TiO_2_ can be enhanced at elevated temperatures up to 100 °C, and that a thin layer of TiO_2_ should be used to avoid surface deactivation [23]. Deactivation of TiO_2_ occurs when intermediate products of acetaldehyde thermal decomposition form at the bottom of a thick layer, where UV light does not reach. In this paper, Au deposited onto TiO_2_ was used for the decomposition of acetaldehyde in synthetic air. Two different methods of Au deposition onto TiO_2_ were used to investigate the impact of Au NPs’ size, distribution, and environment on their catalytic and plasmonic effects. Using the impregnation method, larger Au NPs were expected compared to those obtained by magnetron sputtering. UV and UV-Vis LEDs were used for the process, emitting photons with wavelengths that match the energy of the TiO_2_ band gap and the absorption maximum of Au NPs. LEDs have not been frequently used in photocatalytic processes yet, although they are a very attractive light source compared to others due to their high efficiency, narrow emission spectrum, and low electricity consumption [24,25]. The main purpose of this study was to investigate the impact of the electric field formed near Au NPs and TiO_2_ under light activation (UV or UV-Vis) on acetaldehyde decomposition. It has been reported that the LSPR effect in Au NPs can enhance the formation of superoxide anion radicals, thereby accelerating acetaldehyde decomposition. Moreover, when the process is carried out at 100 °C, the probability of oxygen adsorption on the TiO_2_ surface increases due to the desorption of physically adsorbed water molecules, which can further enhance the formation of superoxide anion radical [23]. We have demonstrated that these superoxide anion radicals are mainly responsible for acetaldehyde decomposition [23]. Although increasing temperature causes decrease in LSPR due to the changes in their dielectric properties, this effect is mainly observed when the temperature rises above 100 °C [26]. As a novelty, both UV and UV-Vis LEDs were used in the process carried out at various temperatures. The mechanism of acetaldehyde decomposition was investigated by in situ FTIR measurements of the photocatalyst surface during the reaction time and light illumination.

## 2. Results

The names of the Au-deposited TiO_2_ samples reflect the gold deposition method and the share of gold in the Au/TiO_2_ composite. For example, “Au/TiO_2__1%” indicates that the material was obtained by heat treatment after impregnation with HAuCl_4_ and that the Au weight fraction is 1%. In contrast, the sample designated Au/TiO_2__S5 was prepared by magnetron sputtering using five sputtering cycles.

### 2.1. SEM Measurements

**Figure 1:**
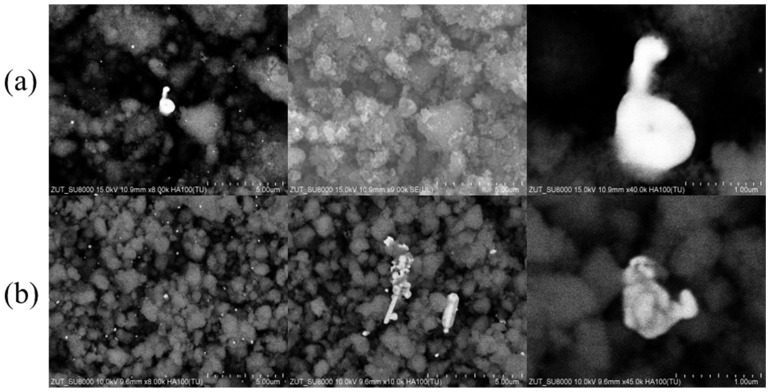
SEM images of (**a**) Au/TiO_2__1% and (**b**) Au/TiO_2__2%.

**Figure 2:**
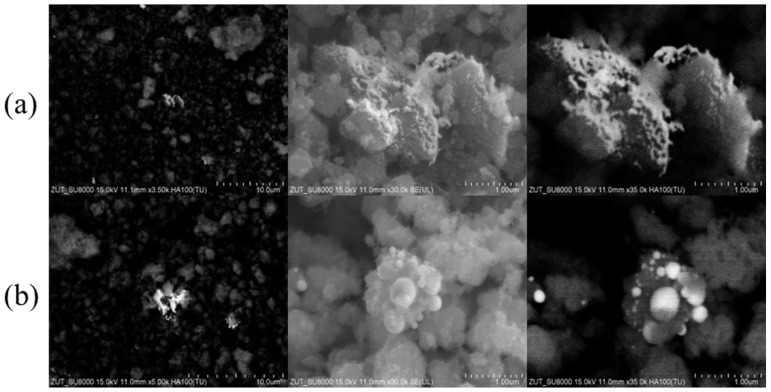
SEM images of (**a**) Au/TiO_2__S3 and (**b**) Au/TiO_2__S5.

The morphology of Au/TiO_2_ samples was investigated by SEM (Figure 1 and Figure 2).

For SEM imaging, two techniques were employed using SE and BSE detectors. The BSE technique produces more contrasting images, allowing easier distinction of gold particles (appearing as brighter areas) from TiO_2_.

The morphology of gold nanoparticles deposited onto TiO_2_ by the impregnation method (as shown in Figure 1) clearly depends on the synthesis conditions and the concentration of the gold precursor. In the Au/TiO_2__1% sample, Au particles showed a shape close to spherical with irregularities, mainly ranging from 60 to 200 nm and a few particles with a diameter above 1 µm (Figure 1a). In the case of sample containing larger amount of gold (2%), the particles of this metal were larger—from 100 nm to about 300 nm (Figure 1b). Moreover, the increased Au content was accompanied by the presence of Au microrods with lengths reaching 2 µm and widths of about 650 nm, as well as Au agglomerates. Based on these observations, it can be assumed that the growth path of Au particles is related to the concentration of HAuCl_4_ and thus to the amount of gold deposited on TiO_2_. A similar effect of increasing Au nanoparticle size with higher concentrations of HAuCl_4_ solution used for TiO_2_ impregnation has been observed by others [18].

The number of Au sputtering cycles on TiO_2_ surface also impacted on the Au particles morphology. The Au/TiO_2__S3 (Figure 2a) sample contains a small number of isolated gold nanoparticles, with sporadic clusters. The Au/TiO_2__S5 sample (Figure 2b) obtained via higher number of Au sputtering cycles shows also some clusters of Au nanoparticles, but with larger sizes, ranging from 70 to 450 nm.

### 2.2. XRD Measurements

**Figure 3:**
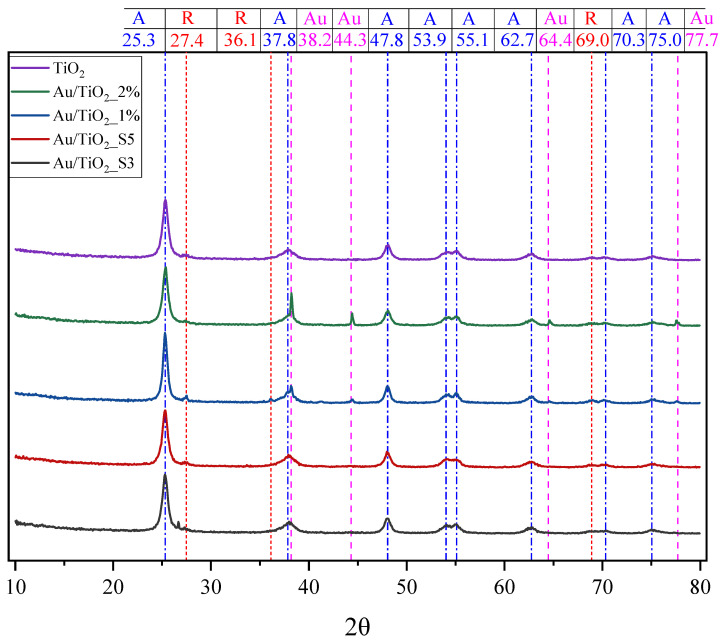
XRD patterns of TiO_2_ and Au/TiO_2_ samples.

The phase compositions of pristine TiO_2_ and Au/TiO_2_ samples was measured by X-ray powder diffraction. Collected XRD patterns are shown in Figure 3.

The diffraction patterns of the samples obtained via impregnation show distinct signals, indicating the presence of both TiO_2_ and metallic gold, as expected. The dominant peak at 2θ = 25.3° corresponds to the (101) plane of TiO_2_ in anatase phase. Additional peaks at 2θ = 27.4°, 36.1° and 69.0° point to the rutile phase of TiO_2_. In turn, the reflections at approximately 2θ = 38.2° (111), 44.3° (200), 64.4° (220), and 77.7° (311) confirm the presence of metallic Au in the Au/TiO_2_ samples obtained by impregnation. The lack of characteristic reflexes from gold in the samples obtained by magnetron sputtering may be related to the uniform dispersion of Au particles on TiO_2_ surface or their small sizes, below 3 nm [27,28,29]. It can be stated that gold deposition did not alter the TiO_2_ crystalline structure, as no peak shifts, broadening, or secondary phases were observed. It means that the Au nanoparticles were adsorbed onto the TiO_2_ surface rather than located at the titania defect sites.

### 2.3. XRFS Measurements

The contents of Au in the Au/TiO_2_ samples were analysed by XRFS method. The results are presented in Table 1.

Performed elemental analysis indicated that the Au/TiO_2__1% and Au/TiO_2__S5 samples had the same gold contents (1.4 wt%), whereas the lowest loading of Au NPs on TiO_2_ was in the Au/TiO_2__S3 sample, below 1 wt%.

### 2.4. UV-Vis/DR Measurements

**Figure 4:**
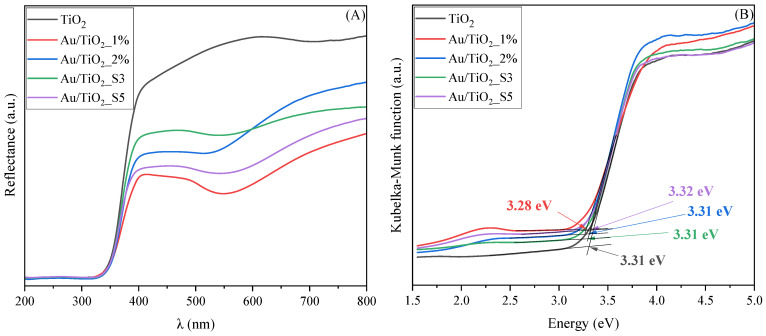
UV-Vis/DR (**A**) and UV-Vis/DR (Kubelka–Munk) (**B**) spectra of TiO_2_ and Au/TiO_2_ samples.

The optical properties of the Au/TiO_2_ and TiO_2_ samples were investigated using UV–Vis/DR spectroscopy. The recorded reflectance spectra (shown in Figure 4A.) were transformed to the corresponding absorption spectra by applying the Kubelka–Munk function (based on the Tauc method) and taking into account that TiO_2_ is an indirect band gap semiconductor. Such transformed spectra were plotted against the photon energy (Figure 4B) in order to determine the energy of the band gap. The *x*-axis intersection point of the linear fit of the Tauc plot gives an estimate of the band gap energy. A detailed procedure for determining the band gap energy of the modified semiconductor photocatalyst is described elsewhere [30].

In the recorded spectra of all gold-modified samples (Figure 4A), a reduced light reflectance is observed in the region of around 500–550 nm, which is typical for Au NPs. Regardless of the amount of gold nanoparticles content in the sample, the band-gap values remain within the narrow 3.28–3.32 eV range. This indicates that the introduction of gold has no important impact on the position of TiO_2_ band edge. Therefore, any enhancement in visible light activity of the Au-modified TiO_2_ samples is expected to come mainly from the LSPR effect of Au NPs or interfacial charge transfer processes rather than from the change in the electronic structure of TiO_2_ [31,32].

### 2.5. Photoluminescence Measurements

**Figure 5:**
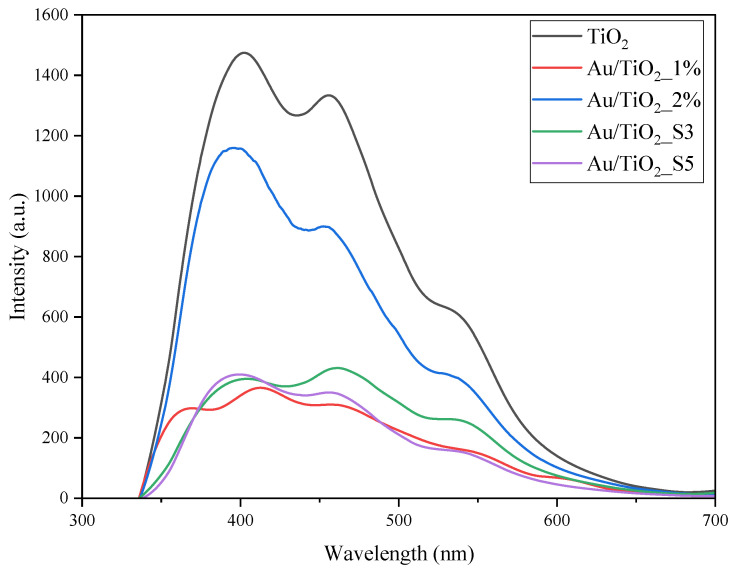
Photoluminescence emission spectra of TiO_2_ and Au/TiO_2_.

Photoluminescence spectra were measured in order to determine the abilities of TiO_2_ and Au/TiO_2_ samples for recombination. The samples were excited with a wavelength of 290 nm, and emission spectra were recorded at the range of 330–700 nm. The results are presented in Figure 5.

All the emission spectra of the Au/TiO_2_ samples revealed lower intensity than that of pure TiO_2_, indicating reduced recombination of free charges occurring in these samples under excitation of UV light. However, the sample with a higher quantity of deposited Au NPs, which partially exhibited a microrod structure (2%), exhibited much less suppression in the recombination process compared to the others. The Au/TiO_2__S3 sample, with somewhat lower content of deposited Au NPs (0.8 wt%) than the others (Au/TiO_2__S5 and Au/TiO_2__1%), which contained 1.4 wt% of Au NPs showed slightly higher intensity of emission spectrum, compared to them. It means that both the quantity of Au NPs and their morphology can affect the charge recombination process.

### 2.6. XPS Measurements

**Figure 6:**
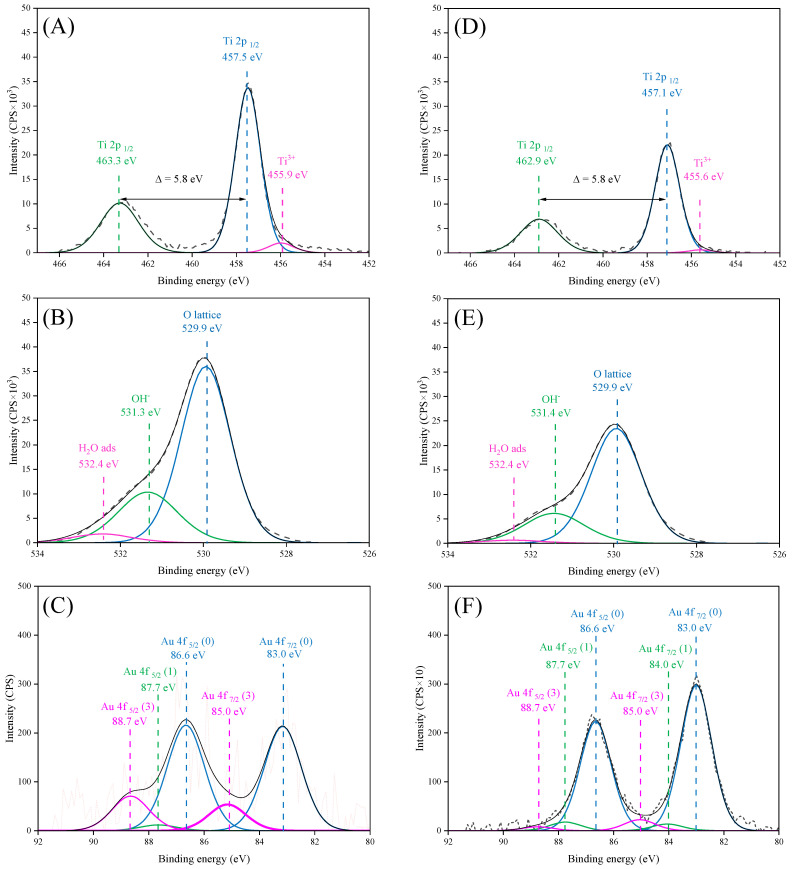
XPS spectra for Au/TiO_2__1%; (**A**) Ti2p; (**B**) O1s; (**C**) Au4f and Au/TiO_2__S5; (**D**) Ti2p; (**E**) O1s; and (**F**) Au4f signals.

The chemical structures of both Au/TiO_2__S5 and Au/TiO_2__1% samples were analysed by the XPS method. In Table 2 and Table 3, there are listed details of their composition and in Figure 6 there is presented XPS spectra for Ti2p, O1s and Au4f signals. These samples were chosen for analysis in order to compare the composition and oxidation states of the gold species obtained on the titania surface through different deposition routes. Both samples contained the same percentage of Au, as confirmed by XRFS analyses.

The reported elemental compositions represent the average values estimated from a sampling depth of approximately 1 nm, based on the assumption of uniform element distribution. However, as this assumption is not strictly valid, the data should be interpreted as approximate. XPS spectra of Ti2p (Figure 6A,D) signals indicated that in both Au/TiO_2__S5 and Au/TiO_2__1% samples, signal comprises two peaks, Ti2p _1/2_ and Ti2p _3/2_. The Ti2p _3/2_ peak is slightly asymmetric due to the presence of Ti^3+^ groups, which are observed following the reduction of titanium. The content of reduced titanium was higher in samples obtained via wet impregnation from HAuCl_4_ solution.

In the case of the O1s region (Figure 6B,E), the spectra were deconvoluted into three distinct components for both samples: the crystal oxygen lattice (Ti-O of TiO_2_), hydroxyl groups (Ti-OH species) and adsorbed molecular water. The most intense peaks are observed in the case of crystal oxygen lattice (529.9 eV). Slightly less intense peak is observed in the case of hydroxyl groups (531.3 eV). In contrast, the peaks with the lowest intensity are observed in the case of adsorbed molecular water (532.4 eV).

The Au/TiO_2__S5 (Figure 6C) sample exhibited three Au4f doublets. The most intense signal originated from metallic gold, as evidence by the presence of signals from Au4f _7/2_ and Au4f _5/2_ orbitals, which exhibited biding energies of 83.0 eV and 86.6 eV, respectively. The signals from gold in the first and third oxidation states are considerably less intense than those of metallic gold. The Au4f _7/2_ and Au4f _5/2_ orbitals corresponding to gold in the first oxidation state were observed to occur at binding energies of 84.0 eV and 87.7 eV, respectively. In the case of gold in the third oxidation state, the Au4f _7/2_ and Au4f _5/2_ orbitals occur with binding energies of 85.0 eV and 88.7 eV, respectively.

Regarding the Au4f region of the Au/TiO_2__1% sample (Figure 6F), the deconvolution allows us to determine three Au4f doublets, corresponding to metallic gold, Au (I), and Au (III) oxidation states. The doublets were located at 83.0 eV and 86.6 eV (metallic Au), 84.0 eV and 87.7 eV (Au(I)), and 85.0 eV and 88.7 eV (Au(III)). Metallic gold was identified as the predominant form in this sample as well [29,33,34,35].

The content of surface gold species was much higher in the Au/TiO_2__S5 sample (2% at.) obtained through the magnetron sputtering method than in the Au/TiO_2__1% sample obtained by impregnation with HAuCl_4_ solution (0.25% at.)

### 2.7. Spectral Characteristics of Irradiance Systems

**Figure 7:**
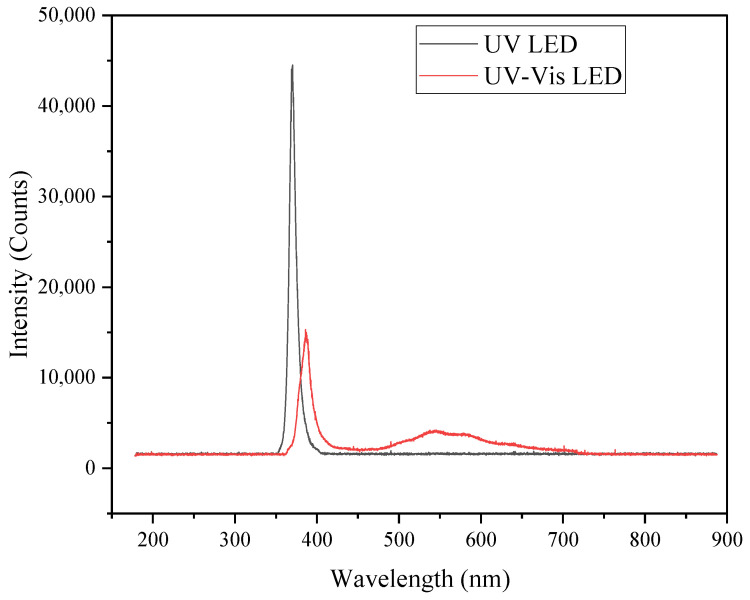
Emission spectra of used light systems/sources.

The radiation intensities of the applied light sources are summarised in Table 4, while their respective emission spectra are displayed in Figure 7.

The UV-LED emits a strong light with a maximum at 365 nm band (approximately 40 Wm^−2^ in the 315–400 nm range) with negligible visible output, whereas the UV-Vis LED provides a weak UV peak with a maximum at 387 nm (0.65 Wm^−2^) with a broad band ranging from 420 up to ~700 nm emission (3.7 Wm^−2^). This enables a comparison to be made between pure UV and UV-Vis, although with significant differences in the irradiance intensities of both systems.

### 2.8. Photocatalytic Decomposition of Acetaldehyde

**Figure 8:**
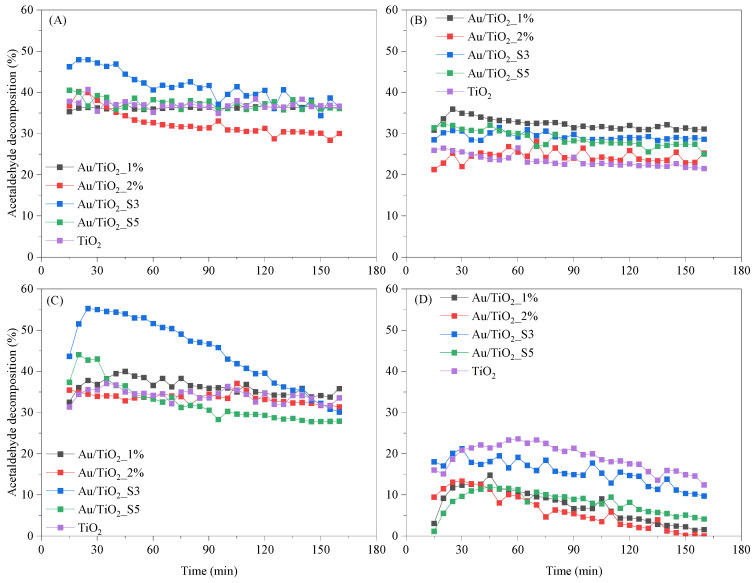
Acetaldehyde decomposition under UV-LED irradiation at (**A**) 100 °C and (**B**) 25 °C, and under UV-Vis-LED irradiation at (**C**) 100 °C and (**D**) 25 °C.

The obtained materials were tested for the removal of acetaldehyde. Figure 7 and Figure 8 show results of the thermo-photocatalytic decomposition of acetaldehyde under UV and UV-Vis LEDs irradiations and in the absence of light. The measurements were performed at both 25 °C and 100 °C.

Under UV-LED irradiation at 100 °C (Figure 8A), the decomposition obtained by using Au/TiO_2__S3 material attains approximately 50% within the first few minutes, but after 40 min its activity gradually decreases to about 35% after 120 min. Au/TiO_2__2% material loses activity during the first 40 min of the test and then performs stably as confirmed by ca. 25% conversion degree. By contrast, Au/TiO_2__1%, Au/TiO_2__S5 and TiO_2_ maintain relatively constant activity as reflected by 30–40% acetaldehyde decomposition throughout the experiment time. Decomposition degree measured for the process carried out at 25 °C under UV-LED irradiation (Figure 8B) does not change notably, however is lower compared to that achieved at 100 °C. When using the material Au/TiO_2__1%, the decomposition of acetaldehyde settles at 30–35%, which is slightly higher than the 20–30% range achieved by Au/TiO_2__2%, Au/TiO_2__S3, Au/TiO_2__S5 and TiO_2_, with minimal fluctuation. When the UV–Vis-LED is employed at 100 °C (Figure 8C), Au/TiO_2__S3 again exhibits the highest initial activity, reaching almost 55% decomposition in the first 20–40 min. Its performance then diminishes steadily until it converges with that of TiO_2_ at approximately 35%. Throughout the same period, Au/TiO_2__1%, Au/TiO_2__2%, and TiO_2_ remain essentially unchanged at 30–40%, whereas Au/TiO_2__S5 displays a slight decline. Under UV-Vis illumination at 25 °C (Figure 8D), TiO_2_ initially shows the greatest activity, but its efficiency decreases progressively over time. The sputter-deposited Au/TiO_2__S3 sample starts at moderate conversion level that diminish during the run. In contrast, the other prepared Au/TiO_2_ samples started with lower activities and continued to decline, ending the two-hour test with less than 5% conversion.

**Figure 9:**
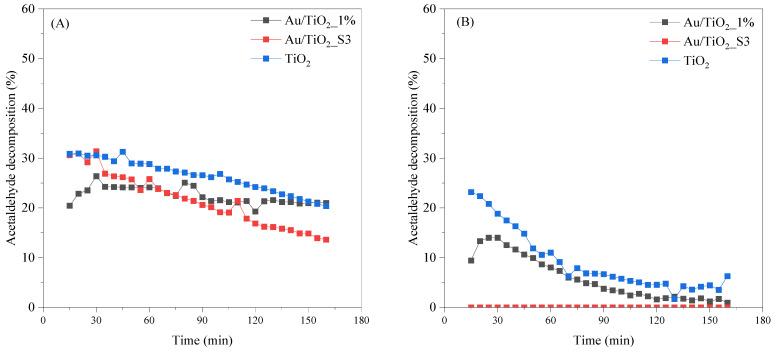
Adsorption and decomposition of acetaldehyde in the absence of light at (**A**) 100 °C and (**B**) 25 °C.

In the absence of light at 100 °C (Figure 9A), acetaldehyde conversion remains well below the levels achieved under UV or UV-Vis irradiations. TiO_2_ exhibits the highest adsorption and thermal decomposition of acetaldehyde, starting at ~30% and declining continuously to ~20% by the end of the run. Au/TiO_2_ follows the same downward trajectory at a slightly lower level, maintaining ~24% for the first 60 min before dropping to ~19 after 180 min. In contrast, the material obtained by sputter coating, Au/TiO_2__S3, deactivates more rapidly ending at around 12%. Reducing the temperature to 25 °C (Figure 9B) nearly suppresses the thermal pathway. TiO_2_ still shows a brief initial response, reaching 20–25% in the first 20 min, but its activity collapses to below 5% by 120 min and remains negligible thereafter. Au/TiO_2__1% shows only a modest peak of ~10% before decaying rapidly to near zero conversion. The sputtered Au/TiO_2__S3 sample remains essentially inactive throughout, with conversion consistently below the detection limit (<1%). It is worth noting that no decomposition occurs at 100 °C in the absence of TiO_2_, effectively ruling out thermal decomposition as a contributing factor under these conditions.

### 2.9. In Situ FTIR Measurements of the Photocatalysts Surface

**Figure 10:**
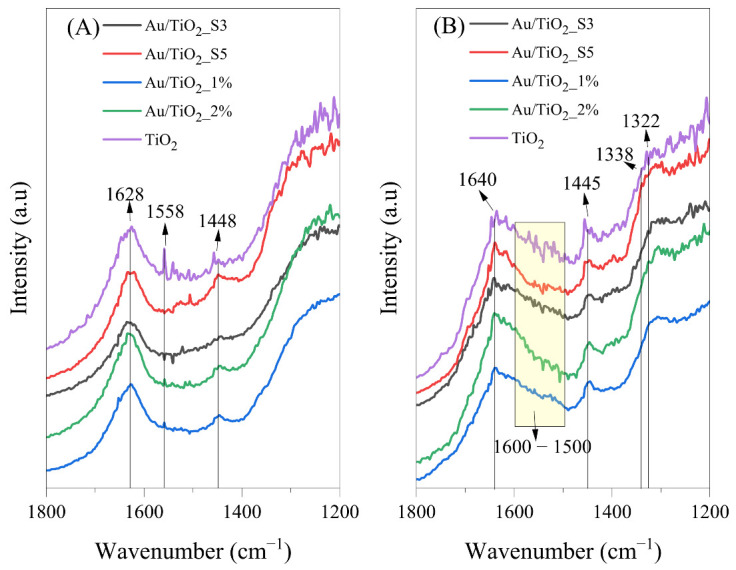
FTIR spectra of bare TiO_2_ and Au-deposited TiO_2_ collected at 100 °C under UV-Vis LED irradiation: (**A**) before and (**B**) after acetaldehyde decomposition.

**Figure 11:**
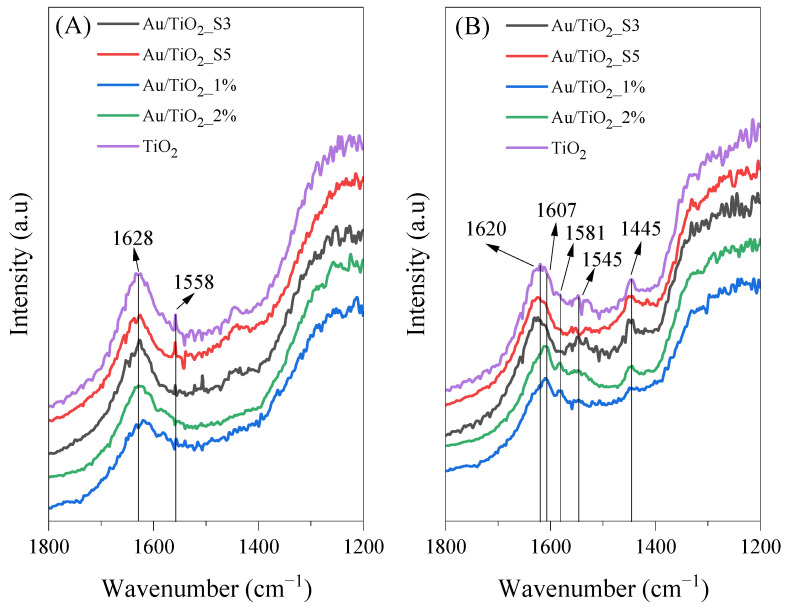
FTIR spectra of bare TiO_2_ and Au-deposited TiO_2_ collected at 100 °C under UV LED irradiation (**A**) before and (**B**) after acetaldehyde decomposition.

**Figure 12:**
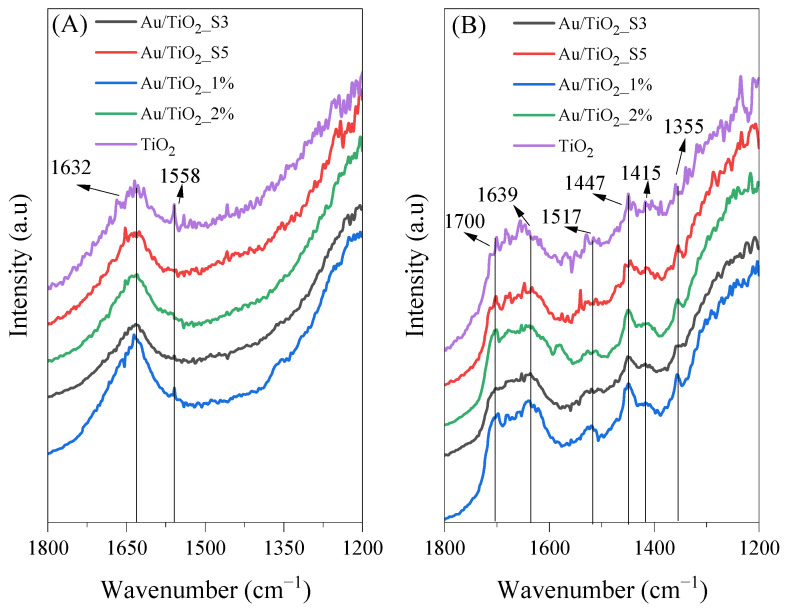
FTIR spectra of bare TiO_2_ and Au-deposited TiO_2_ collected at 25 °C under UV-Vis LED irradiation (**A**) before and (**B**) after acetaldehyde decomposition.

**Figure 13:**
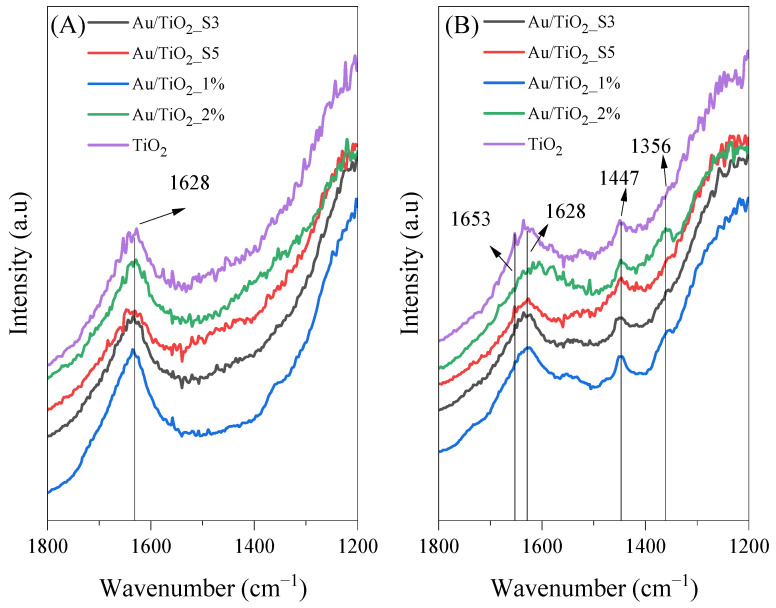
FTIR spectra of bare TiO_2_ and Au-deposited TiO_2_ catalysts collected at 25 °C under UV LED irradiation (**A**) before and (**B**) after acetaldehyde decomposition.

**Figure 14:**
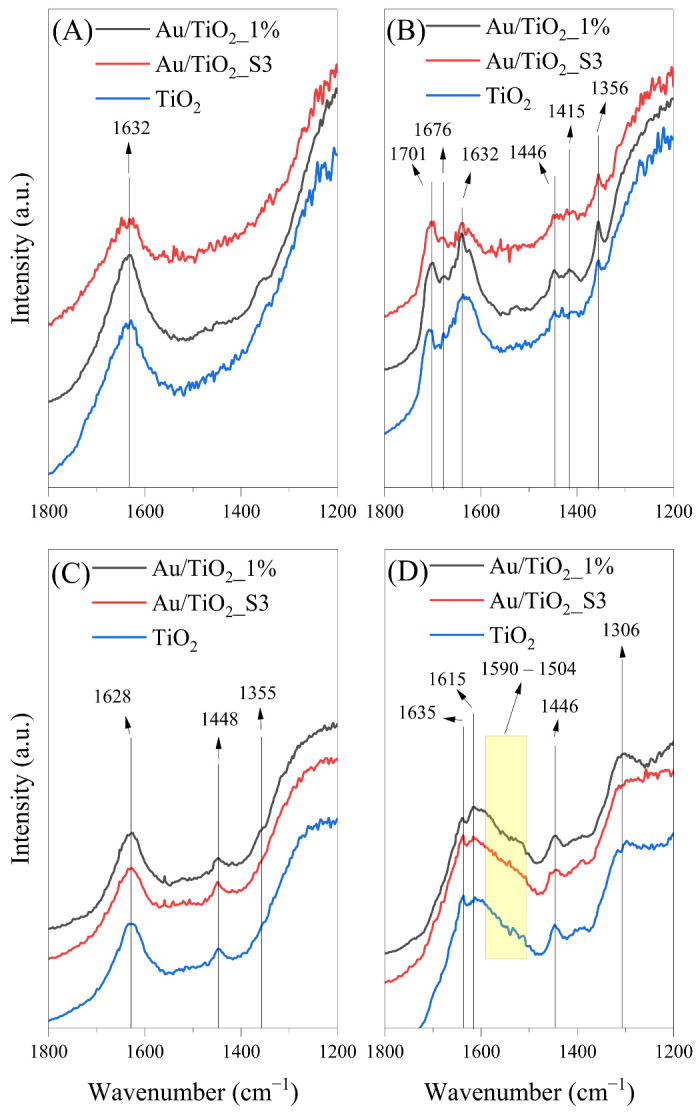
FTIR spectra of Au-deposited TiO_2_ and bare TiO_2_ recorded at the start (**A**,**C**) and end of acetaldehyde decomposition (**B**,**D**) in the absence of light and at 25 °C and 100 °C.

FTIR in situ spectra were collected in order to monitor changes in the photocatalysts surface during the process of acetaldehyde decomposition. Figure 10, Figure 11, Figure 12, Figure 13 and Figure 14 show the FTIR spectra obtained before and after the process conducted under various conditions. The measurements were performed at 25 and 100 °C under UV or UV-Vis LED irradiations, as well as in the absence of light.

In Figure 10, there are presented FTIR spectra of tested samples recorded at the beginning (Figure 10A) and in the end (Figure 10B) of the photocatalytic process conducted under UV-Vis irradiation and at 100 °C. Before irradiation, the bands at 1628, 1558 and 1448 cm^−1^ are observed. The first one can be assigned to the bending vibration of surface hydroxyl groups; the second is associated with bidentate formate species; and the last one may correspond either to the asymmetric deformation vibrations of CH_3_ groups in the adsorbed formic acid or to vibrations of COO^−^ species from CH_3_COO^−^. After the photocatalytic process, new bands appeared in the range of 1600–1500 cm^−1^ that could potentially be assigned to ν_as_(COO) from HCOO^−^ (1594 cm^−1^) and ν_as_(COO) from CH_3_COO^−^ (1558 cm^−1^) [36]. The bands at 1338 cm^−1^ and 1322 cm^−1^ correspond to δ_as_(CH_3_) from adsorbed CH_3_COO^−^ and to δ(CH) from the adsorbed HCOO^−^ species, respectively [1,37].

In Figure 11, FTIR spectra of the same samples used for the acetaldehyde decomposition under UV-LED irradiation and at 100 °C are shown. After the photocatalytic process, new bands are observed at 1581 and 1545 cm^−1^, which can be assigned to the asymmetric stretching vibrations of COO^−^ in the adsorbed formic acid and the adsorbed acetic acid, respectively. Bands at 1445 cm^−1^ may correspond either to the asymmetric deformation vibrations of CH_3_ groups in the adsorbed formic acid or to vibrations of COO^−^ species from CH_3_COO^−^ [36,37].

In Figure 12, FTIR spectra of titania samples collected during photocatalytic process carried out at 25 °C under UV-Vis LED irradiation are shown. Contrary to the process conducted at 100 °C, at room temperature, the titania samples revealed higher surface hydrophilicity and higher intensity of band at 1632 cm^−1^. Moreover, the FTIR spectra recorded in the end of the process revealed major surface transformations. New bands appeared at 1700 cm^−1^, which can be assigned to ν(C=O) from the CH_3_COOH species [36]. Another band at 1639 cm^−1^ was assigned to the bending vibration of surface hydroxyl groups, which slightly shifted from its typical position. A band at 1517 cm^−1^ corresponds to the stretching vibrations of carboxylate groups [38]. The band at 1447 cm^−1^ may correspond either to the asymmetric deformation of δ_as_(CH_3_) or to the symmetric stretching of carboxylate groups *n_s_*(COO). Finally, the band at 1355 cm^−1^ was assigned to s(C-O), originating from adsorbed formate species (HCOO).

In Figure 13, FTIR spectra of the tested samples in the photocatalytic process conducted at 25 °C under UV-LED irradiation are shown. During UV irradiation, some of the carbonyl and carboxylate groups were formed on all the titania samples. A distinct band at 1653 cm^−1^ was attributed to the C=O stretching mode, which is characteristic of adsorbed carbonyl compounds, possibly crotonaldehyde or acetaldehyde [37]. Furthermore, the band at 1447 cm^−1^ was associated with the symmetric stretching vibration of (COO) groups, and the band at 1356 cm^−1^ was linked to the s(C-O) species [1,37].

In Figure 14, FTIR spectra of titania samples collected during flow of acetaldehyde in air at 25 and 100 °C without any irradiation are shown. In the absence of light at 25 °C, several new bands were observed, indicating the formation of surface-bound intermediates. The bands at 1701 and 1676 cm^−1^ were assigned to the C=O stretching vibration of adsorbed acetic acid. The band at 1446 cm^−1^ was attributed to the asymmetric bending vibration of CH_3_, likely originating from crotonaldehyde. The band at 1415 cm^−1^ was assigned to the asymmetric bending vibration of CH_3_ groups associated with adsorbed acetic acid. Additionally, the band at 1356 cm^−1^ was attributed to the C-O stretching mode of the adsorbed formate species, HCOO^−^ [1,36,37,39]

In dark conditions at 100 °C, clear changes in the titania surface, compared to 25 °C, were also observed, suggesting different mechanisms of acetaldehyde decomposition at elevated temperatures. A broad band in the region between 1590 and 1504 cm^−1^ appeared assigned to asymmetric stretching vibrations of carboxylate groups, including both formate and acetate species. A distinct band at 1635 cm^−1^ was observed, likely still related to δ(OH), while the band at 1615 cm^−1^ may correspond to ν(C=C) vibrations from unsaturated aldehydes, such as crotonaldehyde or its hydrogenated derivative, 3-hydroxybutanal. The persistence of the 1446 cm^−1^ band δ_as_(CH_3_) and the emergence of a signal at 1306 cm^−1^ (symmetric CH_3_ deformation) further support the presence of both condensation and oxidation products of acetaldehyde conversion. In Table 5, all the identified FTIR bands with indication of literature references are listed.

## 3. Discussion

**Figure 15:**
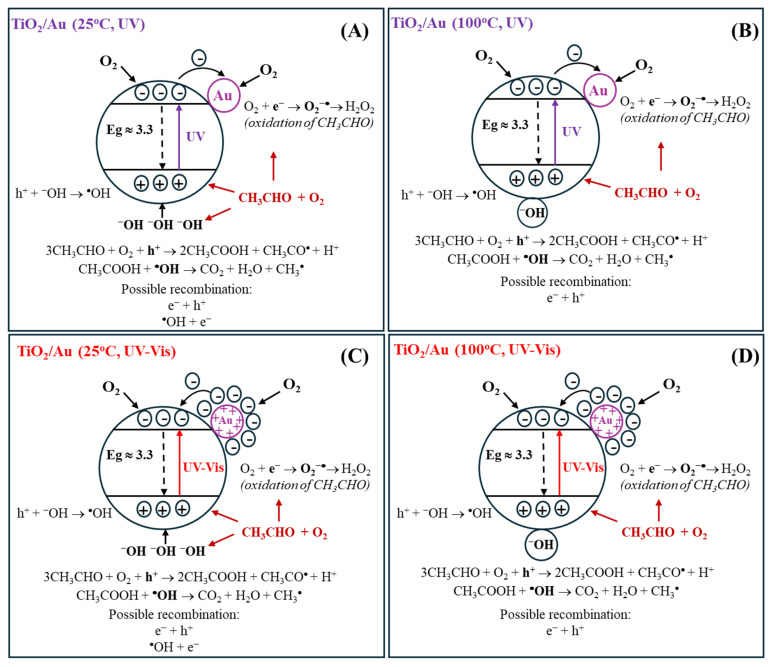
Proposed mechanisms of acetaldehyde decomposition on Au/TiO_2_ under UV irradiation at 25 °C (**A**) and 100 °C (**B**), and under UV-Vis irradiation at 25 °C (**C**) and 100 °C (**D**).

Au NPs were successfully loaded onto the TiO_2_ surface without altering its electronic structure within the band gap. In both deposition methods, higher Au loading led to an increase in particle size due to agglomeration of individual nanoparticles or overlapping caused by repeated magnetron sputtering cycles. In the case of the impregnation method, at higher Au loading on TiO_2_ (2%), SEM images confirmed partial aggregation of Au NPs and formation of microrods. The variety in sizes and structures of the obtained Au particles allowed us to determine their impact on the LSPR effect and its role in enhancing the activity of TiO_2_ towards the decomposition of acetaldehyde. The photoluminescence spectra revealed that at a higher Au deposition level (1.4 wt%) and smaller nanoparticle size, the recombination effect was more pronounced compared to samples with larger Au nanoparticles and microrod structures. A similar impact of the amount of loaded Au NPs on TiO_2_ on charge recombination was observed by others [18], who reported the greatest suppression of the recombination process at a 2 wt% Au NPs. The presence of Au NPs in the oxidised state in the Au/TiO_2__1% sample, as confirmed by XPS, could be beneficial for electron acceptance from the conduction band of TiO_2_ after UV excitation. Therefore, the deposition of Au NPs onto TiO_2_ has been shown to enhance its activity towards acetaldehyde decomposition under UV irradiation. This improvement is attributed to the ability of Au NPs to capture photogenerated electrons, thereby suppressing charge recombination in TiO_2_ (the proposed mechanism is illustrated in Figure 15A). The greatest effect of this process was observed in the Au/TiO_2__1% sample, which had the highest quantity of small Au NPs. The enhancement of acetaldehyde decomposition equalled around 10%. At 100 °C, this effect is less pronounced. The photocatalytic activity of Au/TiO_2_ exposed to UV-LED radiation at a 100 °C was rather comparable to that of TiO_2_, revealing a suppressed effect of Au NPs as electron capture centres. At an elevated temperature, the adsorption of oxygen onto the TiO_2_ surface was probably facilitated by water desorption, which increased the formation of superoxide anion radicals on TiO_2_. It has been reported that the lifetime of charge carriers is longer in a dry TiO_2_ than in hydrated TiO_2_ [41]. Moreover, the higher activity of TiO_2_ with a reduced amount of the adsorbed water molecules toward acetaldehyde decomposition has already been proven [42]. Under these conditions, the role of Au NPs as electron capture centres was less significant. Although the activity of Au/TiO_2__S3 was initially higher than that of TiO_2_, it gradually decreased, eventually reaching (after 150 min of the process) the same activity as TiO_2_. The surface of Au/TiO_2__S3 sample was deactivated faster than that of TiO_2_. FTIR measurements revealed higher intensity band around 1300 cm^−1^ on Au/TiO_2_ samples after photocatalytic process in comparison with TiO_2_, which was attributed to the presence of acetate species. The acetic acid was likely formed during the acetaldehyde decomposition on the surface of Au/TiO_2_ and decomposed slower than on TiO_2_. The suggested mechanism of acetaldehyde decomposition in the presence of Au/TiO_2_ under UV-LED irradiation at 100 °C is shown in Figure 15B.

In the case of UV-Vis-LED radiation used, it was expected that Au NPs would be excited, and due to the LSPR effect [15], an electron cloud would be formed around them. Based on the reports from others [9,16], it was also expected that Au NPs would transfer electrons to the conduction band of TiO_2_, which in turn should result in the increased photocatalytic activity of Au/TiO_2_. However, the results of the conducted experiments at 25 °C indicated the highest activity of the pristine TiO_2_. In general, the activity of all samples under these conditions was reduced due to the lower intensity of UV radiation emitted by the used source. All tested materials showed a gradual decrease in activity. Performed FTIR spectra of the photocatalysts surface after process indicated deposition of some species, such as crotonaldehyde—bands at 1639 cm^−1^ (C=C) and 1447 cm^−1^ (CH_3_); adsorbed acetic acid—bands at 1700 cm^−1^ (C=O) and 1415 cm^−1^ (CH_3_); acetate species—bands at 1517 cm^−1^. All these bands were less intense on TiO_2_ and TiO_2__S3 samples, which showed high activity. This means that acetaldehyde decomposed faster on these samples than on the others. It is stated that activation of Au NPs causes increased adsorption of acetaldehyde (in the form of aldol—crotonaldehyde) on the photocatalyst surface, due to the transport of excited electrons from Au NPs to conduction band of TiO_2_ and formation of holes in the metal nanoparticles. Acetaldehyde adsorption and its transformation to acetic acid proceeded much faster than the decomposition of the reaction products. The scheme of this process is shown in Figure 15C.

At 100 °C, an intensification of acetaldehyde adsorption/decomposition in the initial phase of the photocatalytic process was observed for the Au/TiO_2__S3 sample. The other sample obtained by magnetron sputtering, Au/TiO_2__S5, showed a similar trend; however it was less effective. Most likely, at 100 °C, the interfacial transfer of electrons from Au NPs to TiO_2_ conduction band was enhanced due to the removal of excess water molecules adsorbed on the TiO_2_ surface. In this case, the highest LSPR effect was observed in the Au/TiO_2__S3 sample, which consisted of small sized Au NPs.

Thus, Au NPs deposited on the TiO_2_ surface by magnetron sputtering are strongly activated by visible light. However, the surface of this photocatalyst is deactivated over time under low intensity ultraviolet radiation. The mechanism of acetaldehyde decomposition on Au/TiO_2_ at 100 °C under UV-Vis-LED irradiation is shown in Figure 15D.

It was reported that the loading of Au NPs onto TiO_2_ caused a drastic enhancement of the acetaldehyde adsorption in the dark, and the adsorption amount increased as the Au particle size decreased. Under dark conditions, oxidation of acetaldehyde to acetic acid on Au/TiO_2_ occurred [21]. In our studies, a similar effect was observed.

Acetaldehyde followed the chemical changes in the absence of light and in the presence of TiO_2_ or Au/TiO_2_ and air. After adsorption on the photocatalyst surface, it was mostly converted to acetic acid, as confirmed by FTIR spectra (Figure 14). However, these photocatalyst were rapidly deactivated over time. At 100 °C, the decomposition of acetaldehyde in the dark was more pronounced, but the photocatalysts also experienced deactivation over time. It is worth noting that at higher process temperatures, secondary oxidation products are formed, but they are not stable over time. Deactivation of the photocatalyst is the effect of the lack of full mineralisation of byproducts formed during the conversion of acetaldehyde. Therefore, for stable conversion of acetaldehyde on TiO_2_, UV light of sufficiently high intensity is necessary. The other researchers achieved complete decomposition of acetaldehyde on Au/TiO_2_ samples by applying the subsequent cycles of acetaldehyde adsorption in the dark and then regeneration of the photocatalyst surface by post-heating at 573 K for 2 h [21].

## 4. Materials and Methods

### 4.1. Preparation of TiO_2_

The TiO_2_ was synthesised through a two-step procedure. Initially, amorphous titania (supplied by Grupa Azoty Police, Police, Poland) was hydrothermally treated in an autoclave with use of deionised water at 150 °C for 1 h. After the hydrothermal process, the material was dried at 100 °C to remove residual water. The material was then annealed in a tube furnace at 400 °C under an argon atmosphere for 2 h to obtain the anatase phase.

### 4.2. Preparation of Au-Deposited TiO_2_

Gold deposition was performed using two distinct approaches. In the first method, based on thermal treatment, HAuCl_4_ was prepared by dissolving gold powder (Pol-Aura, Poland 99.9+% purity) in aqua regia, followed by evaporation. Prior to mixing, 1 mL of deionised (DI) water was added to the gold precursor to improve its dispersion. This solution was then added to 1 g of the hydrothermally treated TiO_2_ to achieve a gold content of either 1% or 2% by weight. The resulting mixture was then subjected to the same annealing process (400 °C, argon flow, 2 h), allowing the deposition of metallic gold [43,44] onto the TiO_2_.

In the second method, gold was deposited onto 1.8 g of annealed TiO_2_ using magnetron sputtering. The process was carried out with a Cressington 108 auto plasma sputtering system, applying either 3 or 5 sputtering cycles, each lasting 120 s at a current of 40 mA. Between each cycle, the material was thoroughly stirred to ensure uniform gold distribution across the surface of the TiO_2_ particles.

### 4.3. Analytical Techniques

XRD measurements of Au/TiO_2_ powders were performed using a PANanalytical diffractometer (Almelo, The Netherlands) with a copper X-ray source (λ = 0.154439 nm). The diffraction patterns were recorded over a 2θ range of 10 to 100°, using a step size of 0.013°. The equipment was operated at 35 kV and 30 mA during the analysis.

X-ray fluorescence (XRF) analysis was carried out using an Epsilon3 spectrometer (PANanalytical, Almelo, The Netherlands) to quantify the gold content in the powdered samples.

The surface chemical composition was investigated by X-ray photoelectron spectroscopy (XPS). The measurements were carried out with a commercial multi-purpose ultra-high vacuum (UHV) surface analysis system (PREVAC, Rogów, Poland). A non-monochromatic XPS source was used together with a kinetic energy electron analyser (SES 2002; Scienta, Taunusstein, Germany). The spectrometer was calibrated using the Ag 3d5/2 transition, and Mg Kα radiation (hν = 1253.6 eV) was used as the excitation source for the analysis. The acquired spectra were processed and analysed using CasaXPS software (version 2.3.16).

Photoluminescence spectra were recorded in a fluorescence spectrometer Hitachi F-2500 using a low-temperature sample compartment accessory. The measurements were performed at the temperature of liquid nitrogen, at an excitation wavelength of 290 nm. The emission spectra were recorded in the range of 330–700 nm.

UV-Vis diffuse reflectance (UV-Vis/DR) spectra were obtained using a V-650 spectrophotometer (Jasco International Co., Ltd., Tokyo, Japan). Barium sulphate (BaSO_4_) was used as the reference material. To determine the optical band gap of the samples, the recorded spectra were transformed into the Kubelka–Munk function. Detailed description of this method was reported in the other paper [31].

SEM images of Au-deposited TiO_2_ were obtained using an ultra-high-resolution field emission scanning electron microscope (UHR FE-SEM Hitachi SU8020, Tokyo, Japan).

The radiation intensity of the applied UV radiation was determined using a HD2102.1 photoradiometer (TEST-THERM, Kraków, Poland). Furthermore, a USB4000 spectrometer (Ocean Optics, Inc., Orlando, FL, USA) was utilised to analyse the spectral characteristics of both the UV-LED and UV-Vis light sources.

### 4.4. Photocatalytic Test

Studies of thermo-photocatalytic decomposition of acetaldehyde were performed in a high-temperature reaction chamber (Harrick, Pleasantville, NY, USA). Real-time FTIR spectra were recorded using a Thermo Nicolet iS50 FTIR spectrometer (Thermo, Waltham, MA, USA).

UV irradiation was supplied through a quartz window using two different illumination sources The first used a 365 nm UV LED diode (LABIS, Warsaw, Poland). The second was a custom built system containing both a UV LED and a VIS diode. The distance between the LED and the catalyst bed was maintained at 12 mm.

The gases (acetaldehyde in nitrogen, 300 ppm, and synthetic oxygen, 5.0 grade, both supplied by Messer, Police, Poland) were controlled by mass flow controllers and supplied to the system through two separate inlets. These gases were combined to form a synthetic air mixture (with an acetaldehyde concentration of 240 ppm after mixing). After the thermo-photocatalytic reaction, the gas flow was fed into a gas chromatograph (GC-FID; SRI Instruments, Menlo Park, CA, USA) equipped with an automated sample loop. The concentration of acetaldehyde was determined by analysing the chromatograms.

## 5. Conclusions

This study demonstrates that the deposition of gold nanoparticles (Au NPs) onto TiO_2_ significantly enhances the decomposition of acetaldehyde under UV light irradiation by suppressing electron-hole recombination. Photocatalytic efficiency improves with an increased amount of well-dispersed Au NPs but decreases when larger Au microrods are present.

Under visible light, the localised surface plasmon resonance (LSPR) effect of small, well-dispersed Au NPs promotes the conversion of acetaldehyde to acetic acid. However, catalyst deactivation occurs over time, reducing performance. For instance, at 100 °C under UV-Vis irradiation, the Au/TiO_2_ sample containing 0.8 wt% Au initially achieved a 55% conversion rate of acetaldehyde, which then declined to 30% after 150 min—matching the activity of pure TiO_2_.

Increasing the reaction temperature from 25 °C to 100 °C enhances acetaldehyde degradation, raising conversion rates from 22% to 35% for TiO_2_ and from 32% to 35% for the most active Au/TiO_2_ sample under UV light.

When comparing deposition methods, magnetron sputtering produces smaller, more evenly distributed Au NPs on the TiO_2_ surface. This leads to more effective LSPR activation under visible light than the impregnation method using an HAuCl_4_ solution. The latter tends to yield larger Au NPs with incomplete reduction, which can be beneficial for capturing electrons from TiO_2_.

Overall, Au NP deposition and elevated temperature both contribute to enhanced photocatalytic performance under UV irradiation. However, catalyst deactivation, due to the accumulation of byproducts, limits long-term efficiency under UV-Vis irradiation. Future work should, therefore, explore the use of higher-intensity UV-LED sources to improve by-product mineralisation and investigate closed systems with cyclic adsorption and photocatalytic decomposition to maintain catalyst activity.

## Figures and Tables

**Table 1:** Content of Au in Au/TiO_2_ samples (analysed by XRFS).

Sample	Content/wt% Au
Au/TiO_2__1%	1.4
Au/TiO_2__2%	3.0
Au/TiO_2__S3	0.8
Au/TiO_2__S5	1.4

**Table 2:** Elemental composition of Au deposited TiO_2_ based on XPS spectra.

Sample	Elemental Surface Content [% at.]		
	O1s	Ti2p	Au4f
Au/TiO_2__1%	72.19	27.56	0.25
Au/TiO_2__S5	72.36	25.64	2.01

**Table 3:** Share of surface components in Au deposited TiO_2_, from XPS analysis.

Sample	Ti2p			O1s			Au4f		
	Ti2p _3/2_ [%]	Ti2p _1/2_ [%]	Ti^3+^ [%]	O lattice [%]	^−^OH [%]	H_2_O ads [%]	Au(0) (metallic Au) [%]	Au(1) [%]	Au(3) [%]
Au/TiO_2__1%	66.16	29.94	3.9	71.22	24.54	4.24	75.89	2.10	22.01
Au/TiO_2__S5	66.84	31.27	1.9	74.3	23.28	2.42	89.43	5.35	5.22

**Table 4:** Spectral irradiance of the irradiance systems.

Illumination System	315–400 nm (Irradiance, W/m^−2^)	400–1050 nm (Irradiance, W/m^−2^)
UV-LED	40.00	0.05
UV-VIS LED	0.65	3.70

**Table 5:** Assignment of the FTIR bands.

Molecule	Vibration Mode	Wavenumber (cm^−1^)	References
CH_3_CH=CHCHO	ν(CO)	1653	[37]
H_2_O	*d*(OH)	1620–1630	[40]
HCOO^−^	ν*_s_*(CO)	1355	[1]
CH_3_CH=CHCHO	δ_as_(CH_3_)	1445–1448	[37]
CH_3_COO^−^	*n_s_*(COO)	1444	[36]
HCOO^−^_ad_	ν_as_(COO)	1558	[37]
CH_3_COOH_ad_	ν(C=O)	1700	[37]
CH_3_COOH_ad_	ν(C=O)	1676	[37]
CH_3_CH=CHCHO	*n*(C=C)	1632	[37,39]
CH_3_COOH_ad_	δ_as_(CH_3_)	1412–1415	[1,36,37]
CH_3_COOH	δ_as_(CH_3_)	1306	[37]
CH_3_COO^−^_ad_	δ_as_(CH_3_)	1338	[37]
CH_3_COO^−^	ν(COO)	1517	[38]
HCOO^−^	δ(CH)	1322	[37]
HCOO^−^	ν_as_(COO)	1581	[37]
CH_3_COO^−^_ad_	ν_as_(COO)	1545	[37]

## Data Availability

All published data will be available in the open repository https://mostwiedzy.pl (accessed on 24 July 2025).
